# Evodiamine: A Novel Anti-Cancer Alkaloid from *Evodia rutaecarpa*

**DOI:** 10.3390/molecules14051852

**Published:** 2009-05-18

**Authors:** Junlin Jiang, Changping Hu

**Affiliations:** Department of Pharmacology, School of Pharmaceutical Sciences, Central South University, Xiang-Ya Road #110, Changsha 410078, China; E-mail: snow_jjl@yahoo.com.cn (J-L.J.)

**Keywords:** evodiamine, anti-cancer, apoptosis, proliferation, angiogenesis

## Abstract

Traditional Chinese herbs are regarded as a new and promising source of potential anti-cancer remedies and new chemotherapy adjuvants to enhance the efficacy of chemotherapy and/or to ameliorate its side effects. Extensive investigations have been undertaken both in the experimental and clinical studies over the years to augment the efficacy of chemotherapy. *Evodia rutaecarpa* is a very popular multi-purpose herb traditionally used in China for the treatment of headaches, abdominal pain, postpartum hemorrhage, dysentery and amenorrhea. The major constituents of *Evodia rutaecarpa* are evodiamine and rutaecarpine. Growing evidence demonstrates that evodiamine possesses anti-cancer activities both *in vitro* and *in vivo* by inhibiting proliferation, invasion and metastasis, inducing apoptosis of a variety of tumor cell lines. This review is aimed to summarize the recent researches on evodiamine focusing on anti-cancer activity and to highlight molecular mechanisms during the past ten years.

## 1. Introduction

Chinese herbs have been and still are widely used as important remedies in Oriental medicine. Over the recent years, a variety of biologically active constituents have been isolated from these sources and confirmed to have anti-cancer activity in both experimental and epidemiologic studies. *Evodia rutaecarpa* (Chinese name: Wu-Chu-Yu), has been used for a long time as a traditional Chinese medicine for the treatment of gastrointestinal disorders, headache and postpartum hemorrhage [1,2,3]. Evodiamine, a quinolone alkaloid, is the major component isolated from the fruit of *Evodia rutaecarpa* and has been shown to possess various biological effects, such as testosterone secretion [4], catecholamine secretion [5], antinociceptive [6], anti-inflammatory [7], anti-obesity [8], vasodilatory [9], thermoregulatory [10] and uterotonic effects [11]. Studies concerning the cytotoxicity or inhibitiory activity on cancer cell migration screening of alkaloids have shown that evodiamine exhibits the strongest cytotoxicity activity against human colon and hepatoblastoma cell lines and inhibitory activity on human colon carcinoma cell [12,13]. Further studies demonstrate that evodiamine has anti-tumor potential by inhibiting proliferation, inducing apoptosis and reducing invasion and metastasis of a wide variety of tumor cells, including breast cancer cells [14], prostate cancer cells [15,16,17], leukemic T-lymphocyte cells [18,19], melanoma cells [20], cervical cancer cells [21], colon cancer cells [22] and lung cancer cells [23]. More importantly, evodiamine not only sensitizes chemoresistant breast cancer cells to adriamycin, but also shows little toxicity against normal human peripheral blood cells [14]. The purpose of this review is to summarize the recent progress of research on the anti-tumor mechanisms of evodiamine, which suggests an exciting future for such pursuits in oncology.

## 2. Biological functions

### 2.1. Effects on apoptosis

Apoptosis is an essential and highly conserved model of cell death, which is important for normal development and suppression of oncogenesis. Members of the caspase family and the Bcl-2 family play important roles in inhibiting or promoting apoptosis [24]. The induction of apoptosis in tumor cells is an important mechanism for the efficiency of chemotherapy drugs. Numerous studies demonstrate that evodiamine alters the balance of anti-apoptotic Bcl-2 and proapoptotic Bax protein expression and induces apoptosis by activation of either initiator caspase (caspase-8 and 9) or effector caspase (caspase-3) in a variety of tumor cell lines including human melanoma A375-S2 cells [25,26], acute leukemia CCRF-CEM cells [19], leukemic U937 cells [18], androgen-dependent prostate cancer LNCap cells and androgen-independent prostate cancer PC-3 and DU145 cells [15,16,17], breast cancer NCI/ADR-RES cells [14] and murine fibrosarcoma L929 [27]. Further studies have shown that evodiamine induces apoptosis by both caspase-dependent and caspase-independent pathways. The proapoptotic effect of evodiamine in human cervical cancer Hela cells [21] is completely blocked by Pan-caspase inhibitors, z-VAD-fmk or z-DEVD-fmk, indicating that evodiamine induces cell apoptosis via mitochondrial caspase-dependent apoptotic pathway. It is well known that activation of caspase-independent apoptotic pathway is mediated by the translocation of apoptosis-inducing factor (AIF) into nucleus. Lee *et al.* [18] showed that translocation of AIF into the nucleus is found in human leukemia U937 cells pretreated with evodiamine. In another study, the caspase inhibitor z-VAD-fmk was shown to partially suppress evodiamine-induced apoptosis in A375-S2 cells [25], which further confirms that the caspase-independent cell death mechanism involves in the alternate pathway activated by evodiamine. Moreover, Wang *et al.* showed that evodiamine is able to decrease anti-apoptotic protein S1RT1 expression and enhance p53 expression and phosphorylation in A375-S2 cells by increased expression ratio of Bax/Bcl-2 through inactivation of PI3-K/PKC survival pathway [28].

### 2.2. Effects on cell proliferation and cell cycle

Evidence shows that evodiamine reduces tumor development by inhibiting cancer cell proliferation and altering cell cycle. Studies comparing a number of alkaloidal components of *Evodia rutaecarpa* show that evodiamine inhibits the proliferation of human cancer cell lines representative of acute leukemia [19], malignant melanoma [29], lung cancer [23], prostate cancer [15,16,17], breast cancer [14] and cervical cancer [21] in a dose- and time-dependent manner. An additional study by Ogasawara *et al.* [22] demonstrated that evodiamine causes a 70% reduction in the formation of lung metastases induced by colon carcinoma 26-L5 cells in mice. Compared with controls, those mice administrated evodiamine showed significantly reduced tumor multiplicity. Moreover, evodiamine possesses the strongest anti-proliferation effect on cervical cancer Hela cells among the alkaloidal components of *Evodia rutaecarpa* and compared to 2.4-dihydroxy-5-fluoropyrimidine (5-Fu) at the same concentration [21].

It is well known that suppression of proliferation rates involves cell cycle progression arrest. A search of published studies shows that evodiamine promotes cell cycle arrest at G2/M phase in most cancer cell lines and acts in a time- and dose-dependent manner. NCI/ADR-RES cells exposed to increasing concentrations of evodiamine for 12 h show that evodiamine initiates a concentration-dependent blockage of cell cycle at G2/M phase, and a longer duration of incubation (24 h) leads to a more pronounced arrest at both the sub-G1 phase and G2/M phase [14]. Studies in both androgen-dependent prostate cancer LNCaP cells and androgen-independent prostate cancer DU145 and PC3 cells [15,16,17] also show that evodiamine arrests the cell cycle at the G2/M phase. The cell cycle progression is regulated by activation and inactivation of different classes of cyclins, cyclin-dependent kinase (Cdk) and other regulatory proteins. Among them, the activated Cdc2/Cyclin B complex controls cell cycle progression from G2 phase into M phase [30]. In both androgen-dependent prostate cancer LNCaP cells and androgen-independent prostate cancer DU145 and PC3 cells, the mechanisms of evodiamine-induced G2/M arrest were investigated by assaying the activity of Cdc2. LNCaP cells exposed to evodiamine show a significant enhancement in the protein expression of cyclin B1 and Cdc 2 phosphorylation on Thr 161 site. Cdc2 activation at the onset of mitosis results from the concurrent inhibition of Wee-1 and Myt-1 and activation of Cdc25C phosphatase [31]. Wee-1 and Myt-1 are the negative regulators of Cdc2, which phosphorylates Cdc2 on Thr14 and Tyr15, while activated Cdc25C dephosphorylates Cdc2 on Thy14 and Tyr15 and triggers the activation of Cdc2/CyclinB1 complex [32]. Further analysis in DU145 and PC3 cells reveals that evodiamine not only increases the protein levels of cyclin B1 and phospho-Cdc2 (Thr 161, the active form of Cdc2) in a dose- and time-dependent manner, but that it also diminishes the expression of phospho-Cdc2 (Tyr 15, the inactive form of Cdc2), Myt-1 and unphosphorylated Cdc25C. Together, these results indicate that evodiamine induces cell cycle arrest (G2/M phase) via activation of Cdc2/cyclin B.

### 2.3. Effects on invasion and metastasis

Besides its antiproliferative and pro-apoptotic effects, inhibition of invasion and metastasis are additional mechanisms by which evodiamine halts the cancerous process. Metastasis is a major cause of death in cancer patients. Ogasawara *et al.* [22] showed that evodiamine exhibits suppressive activity on the *in vitro* invasion and lung metastasis of colon 26-L5 cells. The anti-invasive and anti-metastatic effects of evodiamine have been further confirmed on other tumor cell lines, Lewis lung carcinoma (LLC) and B16-F10 melanoma [23]. Evodiamine inhibits the invasion of B16-F10 cells and LLC cells in a concentration-dependent manner with IC_50_ 2.4 μM and 4.8 μM, and achieves 70%-80% suppression at 30 μM in both cell lines. Furthermore, it has been found that evodiamine has little effect on the body weight of tumor-bearing mice at its effective dose, whereas cisplatin produces a serious weight loss [22]. In addition, the effects of the critical structures of evodiamine on tumor cell invasion, migration and metastasis have also been evaluated by comparison with compounds possessing structures similar to that of evodiamine [23]. Results show that functional groups at position 14 and the configuration of hydrogen at position 13b of evodiamine may affect its inhibitory effects on invasion and metastasis of LLC, colon 26-L5 and B16-F10 cells, suggesting that evodiamine may be useful as a promising compound in tumor metastasis therapy. Hepatocyte growth factor (HGF) has been shown to stimulate the invasion, metastasis and migration in a variety of tumor cells. Evodiamine has been proven to reduce HGF-stimulated invasiveness of colon 26-L5, B16-F10 and LLC cells in a concentration-dependent manner and achieve 100% suppression of HGF activity at 30 μM in all of these cell lines [33].

### 2.4. Effects on angiogenesis

Angiogenesis is an important requirement for continued tumor expansion and metastasis. Vascular endothelial growth factor (VEGF) is a key signaling protein involved in angiogenesis, and its overexpression is associated with the process of metastasis. Evodiamine has been seen to directly inhibit human umbilical vein endothelial cells (HUVECs) tube formation and invasion and decrease the protein expression of VEGF and the activation of p44/42 mitogen-activated protein kinase (MAPk, Erk), which is related with endothelial cells angiogenesis. An *in vivo* chicken embryo chorioallanotic membrane (CAM) angiogenesis model has further confirmed the suppression effects of evodiamine on capillary tube formation. Furthermore, evodiamine is able to inhibit lung cancer induced-capillary tube formation of endothelial cells and the release and expression of VEGF. These results suggest a potential role for blockage of angiogenesis and invasion of endothelial cells in evodiamine’s anti-cancer capacity [34].

### 2.5. Other effects

Oxidative stress and inflammatory disorders are now widely known as a major pathogenetic factor of carcinogenic malignant transformation. In A375-S2 cells, evodiamine has been shown to induce oxidative stress and cause subsequent apoptosis by elevating intracellular ROS and nitric oxide levels and reducing cellular antioxidant capacity [35]. The nuclear factor-kappa B (NF-κB) belongs to the transcription factors family and plays a critical role in several signal transduction pathways involved in various cancers. Activation of NF-κB is involved in proliferation, invasion and apoptosis of tumor cells, either promoting or inhibiting, depending on cell type and condition. In human myeloid leukemia cell KBM-5 and lung adenocarcinoma cell H1299 [36], evodiamine inhibits the activation of NF-κB by various carcinogens and inflammatory agents, and causes the reduction of NF-κB -regulated gene products including mediating proliferation (cyclin D1 and c-Myc), antiapoptosis (Survivin and tumor necrosis factor receptor associated factor 1), immunomodulation (chemokines and interleukins) and metastasis (ICAM-1and MMP-9), whereas in human melanoma A375-S2 cells [29], evodiamine activates NF-κB and causes a rapid increase in iNOS expression. 

## 3. Conclusions

The studies described in this review show that the anti-tumor capacity of evodiamine is due to inhibition of proliferation, invasion and metastasis, as well as induction of apoptosis, indicating that evodiamine has the potential to become an effective, systemic anti-tumor remedy. However, the safety, tolerance and pharmacokinetics of evodiamine have not been fully tested on either animals or humans. Some studies have been done to test the acute toxicity of evodiamine in mice (LD_50_, 77.79 mg/kg) [37] and *Drosophila melanogaster* (LD_50_, 3.58 μg per adult) [38], providing a rationale for clinical development of evodiamine as a novel remedy in cancer therapy. Unfortunately, to date, no attempts have yet been made to test the chemotherapeutic potential and safety of evodiamine at the clinical level. In addition, evodiamine is insoluble in water, benzene, or chloroform, very soluble in acetone, and barely soluble in aether or dilute alcohol [39], which means that work on improving the formulation of evodiamine will be necessary and the major challenges in the future are the development of an appropriate dosage form and the evaluation of clinical study employing evodiamine. 

## References

[1] Lee S.H., Son J.K., Jeong B.S., Jeong T.C., Chang H.W., Lee E.S., Jahng Y. (2008). Progress in the studies on rutaecarpine. Molecules.

[2] Yu X., Wu D.Z., Yuan J.Y., Zhang R.R., Hu Z.B. (2006). Gastroprotective effect of fructus evodiae water extract on ethanol-induced gastric lesions in rats. Am. J. Chin. Med..

[3] Wang L., Hu C.P., Deng P.Y., Shen S.S., Zhu H.Q., Ding J.S., Tan G.S., Li Y.J. (2005). The protective effects of rutaecarpine on gastric mucosa injury in rats. Planta Med..

[4] Lin H., Tsai S.C., Chen J.J., Chiao Y.C., Wang S.W., Wang G.J., Chen C.F., Wang P.S. (1999). Effect of evodiamine on the secretion of testosterone in rat testicular interstitial cells. Metabolism.

[5] Yoshizumi M., Houchi H., Ishimura Y., Hirose M., Kitagawa T., Tsuchiya K., Minakuchi K., Tamaki T. (1997). Effect of evodiamine on catecholamine secretion from bovine adrenal medulla. J. Med. Invest..

[6] Kobayashi Y. (2003). The nociceptive and anti-nociceptive effects of evodiamine from fruits of *Evodia rutaecarpa* in mice. Planta Med..

[7] Chiou W.F., Sung Y.J., Liao J.F., Shum A.Y., Chen C.F. (1997). Inhibitory effect of dehydroevodiamine and evodiamine on nitric oxide production in cultured murine macrophages. J. Nat. Prod..

[8] Kobayashi Y., Nakano Y., Kizaki M., Hoshikuma K., Yokoo Y., Kamiya T. (2001). Capsaicin-like anti-obese activities of evodiamine from fruits of *Evodia rutaecarpa*, a vanilloid receptor agonist. Planta Med..

[9] Chiou W.F., Chou C.J., Shum A.Y., Chen C.F. (1992). The vasorelaxant effect of evodiamine in rat isolated mesenteric arteries: mode of action. Eur. J. Pharmacol..

[10] Tsai T.H., Lee T.F., Chen C.F., Wang L.C. (1995). Thermoregulatory effects of alkaloids isolated from Wu-chu-yu in afebrile and febrile rats. Pharmacol. Biochem. Behav..

[11] King C.L., Kong Y.C., Wong N.S., Yeung H.W., Fong H.H., Sankawa U. (1980). Uterotonic effect of *Evodia rutaecarpa* alkaloids. J. Nat. Prod..

[12] Ogasawara M., Matsubara T., Suzuki H. (2001). Screening of natural compounds for inhibitory activity on colon cancer cell migration. Biol. Pharm. Bull..

[13] Xu M.L., Li G., Moon D.C., Lee C.S., Woo M.H., Lee E.S., Jahng Y., Chang H.W., Lee S.H., Son J.K. (2006). Cytotoxicity and DNA topoisomerase inhibitory activity of constituents isolated from the fruits of Evodia officinalis. Arch. Pharm. Res..

[14] Liao C.H., Pan S.L., Guh J.H., Chang Y.L., Pai H.C., Lin C.H., Teng C.M. (2005). Antitumor mechanism of evodiamine, a constituent from Chinese herb *Evodiae fructus*, in human multiple-drug resistant breast cancer NCI/ADR-RES cells *in vitro* and *in vivo*. Carcinogenesis.

[15] Kan S.F., Yu C.H., Pu H.F., Hsu J.M., Chen M.J., Wang P.S. (2007). Anti-proliferative effects of evodiamine on human prostate cancer cell lines DU145 and PC3. J. Cell. Biochem..

[16] Huang D.M., Guh J.H., Huang Y.T., Chueh S.C., Chiang P.C., Teng C.M. (2005). Induction of mitotic arrest and apoptosis in human prostate cancer PC-3 cells by evodiamine. J. Urol..

[17] Kang S.F., Huang W.J., Lin L.C., Wang P.S. (2004). Inhibitory effects of evodiamine on the growth of human prostate cancer cell line LNCaP. Int. J. Cancer.

[18] Lee T.J., Kim E.J., Kim S., Jung E.M., Park J.W., Jeong S.H., Park S.E., Yoo Y.H., Kwon T.K. (2006). Caspase-dependent and caspase-independent apoptosis induced by evodiamine in human leukemic U937 cells. Mol. Cancer Ther..

[19] Huang Y.C., Guh J.H., Teng C.M. (2004). Induction of mitotic arrest and apoptosis by evodiamine in human leukemic T-lymphocytes. Life Sci..

[20] Wang C., Wang M.W., Tashiro S., Onodera S., Ikejima T. (2005). Evodiamine induced human melanoma A375-S2 cell death partially through interleukin 1 mediated pathway. Biol. Pharm. Bull..

[21] Fei X.F., Wang B.X., Li T.J., Tashiro S., Minami M., Xing D.J., Ikeijma T. (2003). Evodiamine, a constituent of *Evodiae Fructus*, induces anti-proliferating effects in tumor cells. Cancer Sci..

[22] Ogasawara M., Matsubara T., Suzuki H. (2001). Inhibitory effects of evodiamine on *in vitro* invasion and experimental lung metastasis of murine colon cancer cells. Biol. Pharm. Bull..

[23] Ogasawara M., Matsubara T., Takahashi S., Saiki I., Suzuki H. (2002). Anti-invasive and metastatic activities of evodiamine. Biol. Pharm. Bull..

[24] Rao L., White E. (1997). Bcl-2 and ICE family of apoptotic regulators: marking a connection. Curr. Opin. Genet. Dev..

[25] Zhang Y., Wu L.J., Tashiro S., Onodera S., Ikejima T. (2003). Intracellular regulation of evodiamine-induced A375-S2 cell death. Biol. Pharm. Bull..

[26] Zhang Y., Wu L.J., Tashiro S., Onodera S., Ikejima T. (2004). Evodiamine induces tumor cell death through two different pathways: apoptosis and necrosis. Acta Pharmacol. Sin..

[27] Zhang Y., Zhang Q.H., Wu L.J., Tashiro S., Onodera S., Ikejima T. (2004). A typical apoptosis in L929 cells induced by evodiamine isolated from Evodia rutaecarpa. J. Asian. Nat. Prod. Res..

[28] Wang C., Wang M.W., Tashiro S., Onodera S., Ikejima T. (2005). Roles of SIRT1 and phosphoinositide 3-OH kinase/protein kinase C pathways in evodiamine-induced human melanoma A375-S2 cell death. J. Pharmacol. Sci..

[29] Yang J., Wu L.J., Tashiro S., Onodera S., Ikejima T. (2008). Nitric oxide activated by p38 and NF-kappaB facilitates apoptosis and cell cycle arrest under oxidative stress in evodiamine-treated human melanoma A375-S2 cells. Free Radic. Res..

[30] Taylor W.R., Stark G.R. (2001). Regulation of the G2/M transition by p53. Oncogene.

[31] Palmer A., Gavin A.C., Nebreda A.R. (1998). A link between MAP kinase and p34 (cdc2) /cyclin B during oocyte maturation: p90 (rsk) phosphorylates and inactivates the p34 (cdc2) inhibitory kinase Myt1. EMBO J..

[32] Booher R.N., Holman P.S., Fattaey A. (1997). Human Myt1 is a cell cycle-regulated kinase that inhibits Cdc2 but not Cdk2 activity. J. Biol. Chem..

[33] Ogasawara M., Suzuki H. (2004). Inhibition by evodiamine of hepatocyte growth factor-induced invasion and migration of tumor cells. Biol. Pharm. Bull..

[34] Shyu K.G., Lin S., Lee C.C., Chen E., Lin L.C., Wang B.W., Tsai S.C. (2006). Evodiamine inhibits *in vitro* angiogenesis: Implication for antitumorgenicity. Life Sci..

[35] Yang J., Wu L.J., Tashino S., Onodera S., Ikejima T. (2007). Critical roles of reactive oxygen species in mitochondrial permeability transition in mediating evodiamine-induced human melanoma A375-S2 cell apoptosis. Free Radic. Res..

[36] Takada Y., Kobayashi Y., Aggarwal B.B. (2005). Evodiamine abolishes constitutive and inducible NF-kappaB activation by inhibiting IkappaBalpha kinase activation, thereby suppressing NF-kappaB-regulated antiapoptotic and metastatic gene expression, up-regulating apoptosis, and inhibiting invasion. J. Biol. Chem..

[37] Yang X.W., Zhang H., Li M., Du L.J., Yang Z., Xiao S.Y. (2006). Studies on the alkaloid constituents of Evodia rutaecarpa (Juss) Benth var. bodinaieri (Dode) Huang and their acute toxicity in mice. J. Asian Nat. Prod. Res..

[38] Miyazawa M., Fujioka J., Ishikawa Y. (2002). Insecticidal compounds from *Evodia rutaecarpa* against *Drosophila melanogaster*. J. Sci. Food Agric..

[39] Chen A.L., Chen K.K. (1933). The constituents of Wu-Chu-Yu (Evodia rutaecarpa). J. Am. Pharm. Assoc..

